# Application of functional genomics to the chimeric mouse model of HCV infection: optimization of microarray protocols and genomics analysis

**DOI:** 10.1186/1743-422X-3-37

**Published:** 2006-05-25

**Authors:** Kathie-Anne Walters, Michael A Joyce, Jill C Thompson, Sean Proll, James Wallace, Maria W Smith, Jeff Furlong, D Lorne Tyrrell, Michael G Katze

**Affiliations:** 1 Department of Microbiology, University of Washington, Seattle, WA, USA; 2 Department of Medical Microbiology and Immunology, University of Alberta, Edmonton, Alberta, Canada

## Abstract

**Background:**

Many model systems of human viral disease involve human-mouse chimeric tissue. One such system is the recently developed SCID-beige/Alb-uPA mouse model of hepatitis C virus (HCV) infection which involves a human-mouse chimeric liver. The use of functional genomics to study HCV infection in these chimeric tissues is complicated by the potential cross-hybridization of mouse mRNA on human oligonucleotide microarrays. To identify genes affected by mouse liver mRNA hybridization, mRNA from identical human liver samples labeled with either Cy3 or Cy5 was compared in the presence and absence of known amounts of mouse liver mRNA labeled in only one dye.

**Results:**

The results indicate that hybridization of mouse mRNA to the corresponding human gene probe on Agilent Human 22 K oligonucleotide microarray does occur. The number of genes affected by such cross-hybridization was subsequently reduced to approximately 300 genes both by increasing the hybridization temperature and using liver samples which contain at least 80% human tissue. In addition, Real Time quantitative RT-PCR using human specific probes was shown to be a valid method to verify the expression level in human cells of known cross-hybridizing genes.

**Conclusion:**

The identification of genes affected by cross-hybridization of mouse liver RNA on human oligonucleotide microarrays makes it feasible to use functional genomics approaches to study the chimeric SCID-beige/Alb-uPA mouse model of HCV infection. This approach used to study cross-species hybridization on oligonucleotide microarrays can be adapted to other chimeric systems of viral disease to facilitate selective analysis of human gene expression.

## Background

Hepatitis C virus (HCV), a blood-borne pathogen belonging to the Flaviviridae family, is a major cause of chronic hepatitis, progressive liver disease and hepatocellular carcinoma [1,2]. The HCV genome is positive-strand 9.6 kb RNA which encodes a single open reading frame (ORF) flanked by 5'and 3' highly structured un-translated regions (UTR) [3]. Translation of the 3,011 amino acid HCV polyprotein is initiated at an internal ribosome entry site (IRES) located within the 5' UTR [3]. The HCV polyprotein is co-and post-translationally cleaved into the viral structural (core and envelope glycoproteins E1 and E2) as well as nonstructural proteins (NS2, NS3, NS4A, NS4B, NS5A and NS5B) by both host signal peptidases and viral proteases.

Recently, an *in vivo *model of hepatitis C virus (HCV) infection was developed involving a SCID mouse carrying a urokinase plasminogen activator (uPA) transgene under the control of the albumin promoter [4-7]. Expression of the uPA transgene in the mouse liver causes a gradual depletion of the mouse hepatocytes. Transplantation of normal human hepatocytes into these SCID-beige/Alb-uPA mice results in animals with chimeric human livers which can then be infected with HCV. This model provides a unique opportunity to study virtually all aspects of the HCV life cycle, including viral entry, replication, and viral kinetics. The chimeric SCID/uPA mouse model also provides a unique opportunity to study HCV-infected animals which have been transplanted with hepatocytes from different donors, facilitating the analysis of host-specific responses to HCV.

Global gene expression profiling, in which the expression levels of thousands of genes are measured in a single experiment, can provide a molecular portrait of cellular events associated with a diseased state. Gene expression studies have been particularly valuable in the study of HCV-associated liver disease, including the identification of specific patterns of gene expression associated with rapidly progressive fibrosis [8], predicting response to IFN therapy [9], and identifying potential markers of HCV-associated liver disease [10,11]. The use of functional genomics to study chimeric systems, such as those used to study HCV and HIV [12-14], is complicated by the potential cross-hybridization of mouse mRNA to the human microarray. It is possible to selectively analyze the expression of human genes by RT-PCR using primers specific for the human sequences but this restricts the analysis to a limited number of genes that have already been established as being interesting. Microarray experiments have the advantage in that no prior knowledge about potential genes/cellular pathways is required. Unfortunately, probe design for oligonucleotide microarrays primarily focuses on limiting cross-reactivity between different genes within the human genome but does not necessarily take into account cross-reactivity between species.

There is an interest in cross-species hybridization for a variety of reasons, including the lack of microarrays for mammalian systems other than human and rodent. Also, evaluation of cross-species hybridization can potentially be used to identify evolutionary conserved mechanisms and pathways of expression control, metabolic pathways or diseases. Cross-species hybridization on human microarrays has already been demonstrated for a number of species, including pig [15,16], bovine [17], feline [18], *Monodelphis domestica *[19] and *Macaca nemestrina *[20]. However, the focus of the majority of these studies was to take advantage of the cross-species hybridization for the reasons outlined above.

The focus of the present study was to determine the feasibility of applying functional genomics to a chimeric tissue. The SCID-beige/Alb-uPA mouse contains a chimeric liver and so tissue used for gene expression studies will have a certain amount of mouse liver mRNA. Only the gene expression in human hepatocytes is of interest as it is only these cells, not the mouse hepatocytes, which are infected with HCV. In addition, the percent of the liver which contains human hepatocytes is variable both between different regions of the same liver and between individual mice. It is important to know not only which probes on the microarray are affected by hybridization of the mouse liver RNA but also to know the minimum percent human which is required in the liver samples to limit this cross-hybridization. Therefore, the goal was not simply to demonstrate cross-hybridization of mouse mRNA on a human oligonucleotide microarray, but to assess the level of this cross-hybridization, identify the genes affected and determine how this affects the gene expression data from human tissue. The approach used to study cross-species hybridization on oligonucleotide arrays can be adapted to other chimeric human-mouse systems to facilitate selective analysis of human gene expression.

## Results and discussion

### Isolation of liver samples with a high ratio of human to mouse RNA

**Figure 1:**
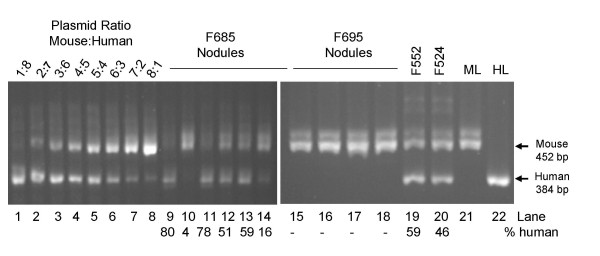
**Determination of the relative amount of human and mouse SCS-alpha genes. **Genomic DNA isolated from dissected chimeric livers (F685, F695, F552, F524) was amplified by PCR with primers specific for SCS-α as described in Materials and Methods. A standard curve was generated using varying rations of human and mouse SCS-α containing plasmids (results shown in lanes 1–8). The percentage of sample which is human is indicated below each lane number. The results of PCR of pure mouse (ML) and human (HL) genomic liver DNA are shown in lanes 21 and 22, respectively.

It has previously been established that the transplanted human hepatocytes develop into red nodules within the mouse liver, which is much paler in color [4]. To obtain liver samples with a relatively high proportion of human RNA, these red nodules were dissected from the chimeric mouse livers. RNA and DNA were isolated from the same sample by sequential Trizol extraction. The relative amount of human and mouse DNA in each sample was determined by semi-quantitative PCR of the single copy gene for the α-subunit of succinyl-CoA synthetase (SCS-α). The primers that were used for PCR were specific for conserved regions in the fourth and fifth exons of human and mouse SCS-α. In mouse SCS-α the fourth intron is 68 bp larger than in human. The results of PCR analysis of two dissected (Figure 1, lanes 9–18) and two whole (lanes 19 and 20) mouse livers are shown in Figure 1. The extra band that is amplified in the mouse samples was also cloned, sequenced and identified as a gene of unknown function found on the X chromosome. The dissection of the liver F685 demonstrated the heterogeneous nature of the chimeric livers, which varied between 4 and 80% human. Typically, samples which contain between 70 and 88% human tissue could be obtained from each mouse.

### Hybridization of mouse liver RNA on human oligonucleotide microarrays in the presence of human liver RNA

While samples containing relatively high percentages of human tissue could be isolated from the chimeric livers, there was still a variable level of contaminating mouse tissue. Therefore, RNA isolated from these samples for use in microarray experiments will contain RNA from mouse hepatocytes. To identify genes affected by mouse liver (ML) RNA hybridization, RNA from identical human liver (HL) samples labeled with either Cy3 or Cy5 was compared in the presence and absence of ML RNA.

A commercially available supply of normal HL RNA was obtained from Ambion. The chimeric mouse model is utilized primarily to study hepatotropic viruses and therefore genes involved in the innate antiviral immune response are of particular interest. To ensure that the cross-hybridization of these genes could be assessed, mouse liver RNA from a transplanted HCV-infected SCID-uPA mouse was used for the microarray experiments. This ML RNA was 100% mouse, based on the PCR assay, and RT-PCR analysis with mouse specific probes confirmed the expression of interferon-regulated genes (data not shown). Table 1 shows the design of each microarray experiment. Genes were selected as being differentially expressed based on 2 criteria, a greater than 95% probability of being differentially expressed (P ≤ 0.05) and a fold change of at least 1.5-fold or 2-fold. Typically, a 2-fold change (P ≤ 0.05) is used to select differentially expressed genes in all microarray experiments [11,21].

**Table 1:** Experimental Design for Microarray Experiments

**Experiment**	**Hybridization Temperature (°C)**	*Channel 1		*Channel 2		**Up-Regulated Genes	
		**HL**	**ML**	**HL**	**ML**	**2-fold**	**1.5-fold**
1	60	1	-	1	-	0	0
2	60	1	1	1	-	1142	2388
3	60	0.5	0.5	1	-	1132	2401
4	60	0.8	0.2	1	-	483	959
5	65	0.8	0.2	1	-	328	646

HL, human liver, ML, mouse liver,

* numbers indicate ug of fluorescent labeled RNA

** The expression ratio for up-regulated genes have a P value ≤ 0.05 based on n = 2 for each experiment and Rosetta Resolver error-model specific for Agilent 22 K Human oligonucleotide microarray.

In the first experiment, HL labeled with either Cy3 or Cy5 fluorescent dye was compared in the absence of any ML RNA. As the human liver samples are identical, the level of differential gene expression between the Cy3 and Cy5 labeled samples should be minimal. As shown in Table 1, this is indeed the case. In total, the expression ratios were measured for 20,163 genes and no genes were selected as being differentially expressed based on both 1.5 and 2-fold changes.

Using this experiment as a base, various amounts of ML RNA labeled with a single dye (either Cy3 or Cy5) was added to the HL versus HL experiment. It was expected that if the mouse RNA does not hybridize to the human oligonucleotide probes, there should be no change in the number of differentially expressed genes. However, if the mouse liver RNA is hybridizing to the probes there should be an increase in the number of differentially expressed genes as the mouse RNA is present in only a single dye. In this way, both the number and the identity of the genes which are affected by ML RNA hybridization can be assessed. Initially, a relatively high amount of mouse RNA was spiked into the HL vs HL experiment, where 50% of either Cy3 or Cy5-labeled RNA was ML RNA. This amount of mouse RNA is substantially higher than what would be considered acceptable for an experiment comparing the gene expression in HCV-infected and naïve animals. Therefore, it should give an indication of the highest level of hybridization that can be expected. This experiment was done in two slightly different ways. In one, the total amount of HL RNA was kept constant: 1 μg HL-Cy3 and 1 μg HL-Cy5 + 1 μg ML RNA labeled with either Cy3 or Cy5. In the other experiment, the amount of RNA for each probe was kept constant: 1 μg HL-Cy3 versus 0.5 μg HL-Cy5 + 0.5 μg ML-Cy5 (or flip dye experiment). This experiment more closely represents how the actual array experiments are done where equal amounts of total RNA are used for each probe, rather than normalizing to the amount of human RNA. However, both experiments were done as it was unclear whether the amount of human RNA labeled for each probe had to be equal to obtain the very small number of differentially expressed genes observed in the HL versus HL experiment. Again, genes were selected as being differentially expressed based on the criteria of at least a two-fold change and P value ≤ 0.05. Unlike the experiment comparing only HL labeled with Cy3 and Cy5, the addition of the ML RNA increased the number of differentially expressed genes substantially. The presence of the ML RNA increased the number of up-regulated genes (2-fold) to either 1142 or 1132 (Table 1), indicating that the mouse RNA was hybridizing to the human oligonucleotide probes. However, even with 50% mouse RNA, the number of genes affected was relatively small, less than 5% of the total number of genes on the slide. Comparison of the two experiments showed a very high correlation coefficient on common signature genes, 0.99 (data not shown).

### Reduction of mouse liver RNA hybridization to human oligonucleotide microarrays

The addition of 50% mouse liver RNA increased the number of up-regulated genes (2-fold) in a HL versus HL experiment from 0 to approximately 1100, indicating that the mouse RNA was likely hybridizing to the oligonucleotide probes. However, samples could be obtained from HCV-infected mice that contain substantially lower percentages of mouse tissue, from 15–30%. To determine if less cross-hybridization would occur in these samples, the previous experiment was repeated with the exception that 20% ML RNA labeled in only a single dye was spiked into the HL versus HL experiment. As shown in Table 1, a substantially smaller number of up-regulated genes (2-fold), 483, were observed in this experiment.

In an attempt to further reduce the amount of ML-RNA hybridization, the specificity of the reaction was increased by using more stringent hybridization conditions. Increasing the hybridization temperature from 60°C to 65°C further reduced the number of up-regulated genes (2-fold) to 328 (Table 1). The two experiments were compared to ensure that the hybridization of the human liver RNA was not significantly affected by the higher temperature. The correlation coefficient on common signature genes is very high, 0.97, indicating that the effect on human RNA hybridization was minimal (data not shown). In addition, comparison of up-regulated genes showed that the majority of the genes are common to both experiments (data not shown), further suggesting that these up-regulated genes were caused by cross-hybridization of ML- RNA.

### Validation of gene expression changes for genes known to be affected by hybridization of mouse liver RNA

**Figure 2:**
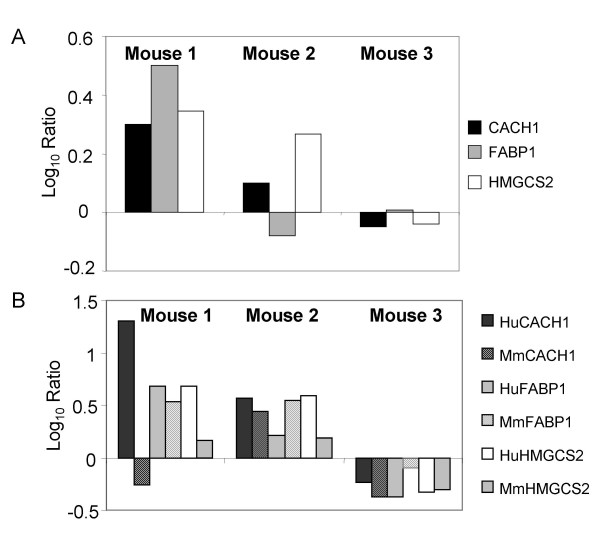
**Expression of lipid metabolism genes in human and mouse liver tissue. **A. Comparison of gene expression levels of lipid metabolism genes by either **A**. Microarray analysis or **B. **Real-time PCR analysis using either human (solid bars) or mouse (dotted bars)-specific probes. Data is shown as log_10 _ratio and reflects the difference in expression between donor-matched HCV-infected and naïve liver tissue. Donor-matched samples contained approximately the same percentage of human tissue. The three HCV-infected mice were transplanted with hepatocytes from different donors.

Real-time quantitative RT-PCR using species-specific probes can differentiate whether the gene expression change observed by microarrays is the result of viral-induced changes in human hepatocytes or simply hybridization of mouse liver RNA. Three lipid metabolism genes, CACH1, FABP1, and HMGCS2, shown to be affected by hybridization of ML-RNA on the human arrays were chosen to determine whether the gene expression changes measured on the arrays was originating from the human tissue or the mouse tissue. The species specificity of each probe was first confirmed by performing real time quantitative PCR analysis of commercially available human and mouse liver RNA samples (data not shown). These probes were then used to determine the expression levels of these genes in HCV-infected chimeric samples relative to donor-matched uninfected samples and the results compared to the data obtained from the corresponding microarray experiments. For both the microarray experiments and quantitative RT-PCR, the expression of these genes in HCV-infected animals was compared to the respective uninfected controls. The microarray experiments showed increased expression of these genes in Mouse 1 and, to a lesser extent, Mouse 2 (Figure 2A). Mouse 3 showed decreased expression of CACH1 and HMGCS2 and essentially no change in FABP1 (Figure 2A). Quantitative RT-PCR demonstrated a change in expression of these genes in both the mouse and human liver tissue, although in general the change was more dramatic in the human tissue (Figure 2B). It is possible that increased the mouse hepatocytes are simply responding to increased lipid/cholesterol released from the HCV-infected human hepatocytes. Importantly, the results obtained using the human-specific probes generally demonstrated a good correlation with the gene expression data from the microarrays, although the ratios calculated from quantitative RT-PCR generally exceeded those obtained using microarrays. This indicates that the cross-hybridization of the ML-RNA is not adversely effecting the gene expression data generated using the microarray, at least with respect to the three genes analyzed.

#### Bioinformatics analysis

To identify potential cross-hybridization candidates in silico, 20,143 Agilent Human 1A (V2) 60 mer oligonucleotide DNA sequences were searched using NCBI BLASTN [26] for similar sequences within a 26,940 NCBI mouse RefSeq [27] DNA sequence database, selecting the best sequence match for each 60 mer with a BLAST expectation value threshold of 10-3. 6,410 mouse RefSeq transcripts representing 5,839 distinct genes showed significant identity to the 60 mer human probes. Average alignment length for the 6,410 matches was 51/60 base pairs with average identity of 92 percent. Of the 1124 genes showing 2-fold differential regulation at P value ≤ 0.05 with 50% mouse RNA, 789 matched mouse RefSeq transcripts using the above BLAST parameters with average alignment length of 53/60 base pairs and average identity of 94 percent. Of the 483 genes showing 2-fold differential regulation at P ≤ 0.05 with 20% mouse RNA, 375 matched mouse RefSeq transcripts with an average alignment length of 53/60 base pairs and average identity of 94 percent.

## Conclusion

Chimeric systems are extremely valuable tools to study human viral disease. They do, however, present a challenge for the use of genomics. While many studies have examined cross-species hybridization on multiple array platforms, including oligonucleotide microarrays [22-24], none have investigated the effect of this cross-species hybridization on gene expression studies using chimeric tissue. The results of the current study demonstrate that functional genomics can be applied to the HCV-infected chimeric SCID-beige/Alb-uPA mouse model and outline a series of experiments that can be adapted to study the level of cross-species hybridization in any chimeric tissue, thus facilitating the use of genomics in these model systems. While mouse liver mRNA from the SCID-beige/Alb-uPA mouse did hybridize to the human oligonucleotide microarray, the number of genes affected was relatively small even when high percentages of mouse liver RNA was present. This hybridization could be reduced by increasing the stringency of the hybridization and also by using liver samples that contained at least 80% human tissue. Furthermore, species-specific quantitative RT-PCR demonstrated that the hybridization of mouse RNA, in general, did not significantly affect the human gene expression measurement obtained from the microarray analysis, at least with respect to the three genes analysed. However, it is likely still important to use RT-PCR to validate the expression level of known cross-hybridizing genes as the extent of hybridization may vary between different probes.

Another way of examining cross-hybridization of the mouse liver mRNA would be to apply the mouse mRNA to the microarray in the absence of human mRNA. However, the purpose of this study was to determine the feasibility of performing gene expression on chimeric tissue. It was not simply to demonstrate that mouse liver RNA would hybridize to human oligonucleotide microarray, but rather to determine how extensive the hybridization was in the presence of excess human RNA. In addition, the platform of microarray used for these studies was a two channel system, meaning that both the reference and experimental samples are hybridized on the same microarray. As such, it generates ratio, rather than intensity, gene expression data which makes it difficult to determine simply the presence or absence of a transcript.

It is important to note that this gene set was derived experimentally and so it is possible that additional genes which are not expressed in the liver will also be affected by hybridization of mouse RNA. Previous attempts, with other array platforms, have been made to use bioinformatics methods to "mask" or eliminate those probes from further analysis which exceed a threshold of mismatching base pairs between a probe and its corresponding transcript sequence [25]. Our analysis shows only a very small difference in average alignment length and percent identity between genes showing significant differential regulation with ML spike-ins and genes matching probes in general. This emphasizes the importance of identifying the genes affected by cross-hybridization experimentally as opposed to using bioinformatics methods. While the experiments outlined in this study were conducted specifically to study virus-host interactions using the SCID-beige/Alb-uPA mouse model of HCV infection, they can be adapted to study the level of cross-species hybridization in any chimeric tissue, thus facilitating the use of genomics in these model systems.

## Methods

### Dissection of mouse livers and isolation of RNA and genomic DNA

All mice were treated according to Canadian Council on Animal Care guidelines. SCID-beige/Alb-uPA mice were transplanted with human primary hepatocytes and then infected with HCV as described previously [4,6]. Mice were euthanized by cervical dislocation and the livers were excised, dissected into small pieces and then snap frozen in N_2(l)_. Total RNA was then isolated using a standard Trizol procedure. Genomic DNA was isolated from the interphase and phenol/chloroform phase according to the manufacture's specifications (Invitrogen). Both RNA and genomic DNA were isolated from the same samples.

### PCR of human and mouse genomic DNA

Genomic DNA from the liver of a SCID-beige/Alb-uPA mouse containing human hepatocytes was amplified by PCR with primers specific for the fourth and fifth exons of the gene for the Succinyl-CoA synthetase alpha subunit (SCS-α). The PCR conditions were: 0.01% (w/v) gelatin, 50 mM KCl, 1.5 mM MgCl_2_, 10 mMTris pH8.3, 0.2 mM each of dATP, dGTP, dCTP, dTTP, 2.5 U of Taq polymerase, and 0.25 μM of each of 5' ttgtgaatatggccaggcatg, (SCS-ex5) and 5' caggagcaacggcttctgtc (SCS-ex4). The underlined nucleotide denotes the position of the nucleotide that is present in the human sequence but not in the mouse sequence. The PCR cycles were: 5 minutes at 95°C, followed by 30 cycles of (30 seconds at 95°C, 30 seconds at 45°C, 30 seconds at 72°C). The resulting PCR products were separated by electrophoresis in a 1.5% agarose gel, excised, purified using a Qiaquick gel extraction kit (Qiagen), cloned into pCR4 TOPO according to manufactures specifications (Invitrogen) and sequenced. The cloned fragments from mouse and human were 452 bp and 384 bp, respectively. For measurement of the relative amount of human and mouse SCS-α the PCR conditions were as follows: 1.2 μg of genomic DNA, 20 mM Tris pH 8.4, 50 mM KCl, 2 mM MgCl_2_, 0.2 mM each of dATP, dGTP, dCTP, dTTP, 2.5 U of Taq polymerase, and 0.25 μM of each of SCS-ex4 and SCS-ex5. PCR cycles were: 10 minutes at 95°C followed by 40 cycles of (45 seconds at 95°C, 45 seconds at 68°C, 45 seconds at 72°C) and a final extension of 10 minutes at 72°C. The cloned plasmids were used as standards, which were varied between 3.5 pg human: 28 pg mouse and 28 pg human: 3.5 pg mouse per reaction. To determine the relative amount of human SCS-α gene in the sample, the PCR products were separated by electrophoresis using a 2.5% agarose gel and then stained with ethidium bromide. The bands were quantified, a correction was made for the size difference, and the results were plotted on a standard curve generated by the plasmid controls.

### Expression microarray format and data analysis

Microarray format, protocols for probe labeling, and array hybridization are described at . Human and mouse liver RNA were labeled separately and then mixed prior to hybridization. Human 1A Oligo Microarray (V2), which contains over 20,000 60-mer probes corresponding to over 18,000 human genes, were purchased from Agilent. Briefly, a single experiment comparing two mRNA samples was done with four replicate Human 1A (V2) 22 K oligonucleotide expression arrays (Agilent Technologies) using the dye label reverse technique. This allows for the calculation of mean ratios between expression levels of each gene in the analyzed sample pair, standard deviation and P values for each experiment. Spot quantitation, normalization and application of a platform-specific error model was performed using Agilent's Feature Extractor software and all data was then entered into a custom-designed database, Expression Array Manager, and then uploaded into Rosetta Resolver System 4.0.1.0.10 (Rosetta Biosoftware, Kirkland, WA) and Spotfire Decision Suite 7.1.1 (Spotfire, Somerville, MA).

### Quantitative RT-PCR

Quantitative real-time PCR (rtPCR) was used to validate the gene expression changes. Total RNA samples were treated with RNAse-free DNase Treatment and Removal Reagents (Ambion, Austin, TX). Reactions were performed in quadruplicate on the ABI 7500 Real Time PCR System (Applied Biosystems, Foster City, CA), using TaqMan chemistry with primer and probe sets selected from the Assays-on-Demand product list (Applied Biosystems) and two endogenous controls, GAPDH and 18S ribosomal RNA. Quantification of each gene, relative to the calibrator, was calculated by the instrument, using the equation: 2-ΔΔCT within the Applied Biosystems Sequence Detections Software version 1.3.

### 

## Abbreviations

SCID, severe-combined immunodeficiency, PBMC, peripheral blood mononuclear cells, HCV, Hepatitis C virus, uPA, urokinase plasminogen activator, SCS-α, succinyl-CoA synthetase, Cy, cyanine, CACH1, cytosolic acetyl-CoA hydrolase, FABP1, fatty acid binding protein 1, HMGCS2, HMG-CoA synthetase 2

## Authors' contributions

KW participated in the design of the study, carried out microarray experiments and drafted the manuscript. MJ participated in sample preparation and characterization, JC conducted the quantitative RT-PCR analysis, MS, SP and JF participated in experimental design. JW performed the sequence alignments and bioinformatics analysis. LT and MK coordinated the study and helped to draft the manuscript.
